# Does tocilizumab have an effect on the clinical outcomes in COVID-19 patients? A meta-analysis of randomized control trials

**DOI:** 10.1186/s40545-023-00662-w

**Published:** 2023-11-20

**Authors:** Faezeh Ghaempanah, Maziar Nikouei, Mojtaba Cheraghi, Arman Jahangiri, Yousef Moradi

**Affiliations:** 1 Student Research Committee, Kurdistan University of Medical Sciences, Sanandaj, Iran; 2 Department of Emergency Medicine, School of Medicine, Besat Hospital, Hamadan University of Medical Sciences, Hamedan, Iran; 3 Social Determinants of Health Research Center, Research Institute for Health Development, Kurdistan University of Medical Sciences, Sanandaj, 66179-13446 Iran

**Keywords:** Tocilizumab, Actemra, COVID-19, Mortality, Systematic review, Evidence synthesis

## Abstract

**Background:**

This meta-analysis was conducted to investigate the impact of tocilizumab on clinical outcomes associated with COVID-19.

**Methods:**

A comprehensive search was conducted across Scopus, PubMed (Medline), Cochrane Library, EMBASE (Elsevier), ClinicalTrials.gov, and Web of Sciences to identify pertinent studies published until May 2022. The primary search terms included "tocilizumab" and "COVID-19". Following the formulation of the search strategy, all identified studies were screened, and the data extraction process was initiated. Subsequently, the Cochrane risk of bias checklist was employed to evaluate the risk of bias. The effects of tocilizumab were assessed utilizing the pooled risk ratio (RR) and the fixed effect model in STATA (version 17).

**Results:**

In this meta-analysis, we analyzed 17 clinical trial studies to assess the impact of tocilizumab on mortality in patients with COVID-19. The pooled risk ratio (RR) for mortality was 0.93 (RR: 0.93; 95% CI: 0.86, 1.00; *I*^2^: 72.39%; *P* value: 0.001). The findings indicated that tocilizumab use was associated with a 4% increase in ICU hospitalization (RR: 1.04; 95% CI: 0.90, 1.20; *I*^2^: 0.00%; *P* value: 0.65). Additionally, tocilizumab administration was linked to a 2% reduction in the requirement for a ventilator (RR: 0.98; 95% CI: 0.90, 1.08; *I*^2^: 26.87%; *P* value: 0.16).

**Conclusion:**

The administration of tocilizumab during the COVID-19 pandemic, prescribed to patients with the virus, exerted a noteworthy impact on reducing outcomes associated with COVID-19.

## Background

More than 2 years have passed since the onset of the severe acute respiratory syndrome coronavirus-2 (SARS-CoV-2), and its pandemic [1]. Based on the World Health Organization (WHO) dashboard, over 500 million confirmed cases, and more than six million deaths due to the COVID-19 disease have been reported up to April 24, 2022 [2]. Although most patients with COVID-19 develop mild to moderate forms of the disease, approximately 15% develop its severe forms which require oxygen support, and around 5% have critical conditions with complications such as respiratory failure [3]. The cytokine release syndrome (CRS) is thought to be an important cause of death in COVID-19 patients, in which IL-6 plays an important role [4, 5]. Interleukin 6 is an inflammatory cytokine with various effects such as inducing synthesis of acute phase proteins like C-reactive protein and serum amyloid A, antibody production by B cells and development of T helper 17 [6]. Increased levels of interleukin 6, and C-reactive protein correlate with mortality related to the COVID-19 disease [7]. Tocilizumab is a monoclonal antibody against interleukin-6 receptor-alpha that has been used for cure of autoimmune diseases particularly refractory rheumatoid arthritis over the past twenty years [8, 9], and is currently under investigation as a treatment option for the COVID-19-related CRS [10]. Recent studies show Tocilizumab reduces the risk of mechanical ventilation in hospitalized patients with severe COVID-19, and also decreases the risk of poor outcomes, and secondary infections in hospitalized COVID-19 patients [11–13]. Previous evidence synthesis studies have investigated the efficiency, safety, and all-cause death of this drug in patients with COVID-19, but no executive study investigating the whole outcomes of tocilizumab in COVID-19 patients including death has been done yet [14–16]. Evidence from non-interventional trials, and open label studies has been contradictory, and no definitive result has been achieved from previous randomized, double-blind, and placebo-controlled trials [12, 17]. During the pandemic, tocilizumab was used in combination with corticosteroids for the treatment of patients with severed forms of COVID-19, who needed mechanical ventilators in the intensive care unit (ICU). Therefore, it is considerable to evaluate the effect of this drug on COVID-19 mortality rather than other outcomes.

This study contributes to the existing knowledge by meticulously investigating the impact of tocilizumab on COVID-19-related outcomes, utilizing a robust methodology involving a comprehensive review of randomized clinical trials up to May 2022. The inclusion of 17 interventional studies and a substantial cohort, with 1936 patients in the tocilizumab group and 2530 in the comparison group, strengthens the study's analytical rigor. The significance of this study lies in its focus on the potential of tocilizumab to mitigate adverse outcomes associated with COVID-19. While there have been numerous systematic reviews and meta-analyses conducted during the same time period, this study addresses a specific gap by honing in on key aspects that may not have been thoroughly explored in the existing literature. The distinctiveness of our approach, coupled with the substantial dataset, aims to provide nuanced insights and fill potential gaps in understanding the efficacy of tocilizumab in the context of COVID-19. Therefore, this study adds a valuable layer of specificity and depth to the current knowledge landscape.

## Methods

The present review was conducted employing a structured methodology comprising six distinct steps, namely the formulation of search syntax and search strategy, screening, selection, data extraction, quality assessment, and meta-analysis. This systematic inquiry adhered rigorously to the guidelines outlined in the Preferred Reporting Items for Systematic Reviews and Meta-Analyses statement (PRISMA) [18].

### Search strategy and information sources

The search encompassed pertinent databases, namely PubMed (Medline), Scopus, Web of Sciences, EMBASE (Elsevier), ClinicalTrials.gov, and the Cochrane Library. The exploration period spanned from the initiation of the COVID-19 pandemic to May 2022. To identify synonyms, the primary study keywords, "tocilizumab" and "COVID-19", underwent an exhaustive process, consulting Mesh, Thesauruses, and Emtree to formulate and tailor search syntaxes for each database. Subsequently, a manual search was undertaken by scrutinizing the references of relevant and ultimately selected articles. In the subsequent phase, duplicate publications were meticulously eliminated based on titles, authors, and publication year, employing Endnote software version 9.

### Eligibility criteria and selection process

The screening process adhered to stringent inclusion criteria, specifically focusing on studies structured according to the PICOT framework. Inclusion criteria stipulated that the study population (P) must comprise individuals with COVID-19, the intervention (I) must involve tocilizumab, the comparison (C) must be made with either a placebo or other medications, and the intended outcome (O) must pertain to the measurement of cases or the percentage of death attributable to COVID-19. Furthermore, the type of studies (T) considered for inclusion were those of an interventional nature, with or without randomization. Studies deviating from this framework were excluded from the meta-analysis. The screening process involved a thorough evaluation of titles, abstracts, and full texts, with both authors independently executing all stages of the search strategy and article screening to ensure a comprehensive and unbiased approach.

## Data collection process and data items

During this phase, essential information was meticulously extracted from the identified studies, encompassing authors' names, publication year, study country, study type, characteristics of the study population, drug dosage and administration protocols, underlying health conditions, age distribution, body mass index data, and specified outcomes (comprising mortality, ventilator requirement, and ICU hospitalization). The data extraction process was executed independently by two authors, namely FGH and MCH. In cases where discrepancies arose, a third party, YM, was engaged to arbitrate and resolve any disputes, ensuring a consensus-driven and objective approach to the data extraction process. This rigorous and collaborative methodology aimed to enhance the reliability and accuracy of the extracted information, laying a foundation for robust subsequent analyses.

### Risk of bias

The quality of study design, sampling strategy, and measurement (reporting assessment) was assessed based on the Consolidated Standards of Reporting Trials (CONSORT) statement [19, 20], and Cochrane Risk of Bias tool. Special issues regarding the cross-over design in 2 of the trials were addressed according to the Cochrane handbook of systematic reviews. Two authors (FGH and MN) independently assessed the risk of bias in Revman 5.3 software within each trial [21]. Any disagreement was resolved by consensus, and by consultation with a third party (YM) in case of persistent disagreement.

### Synthesis methods and effect sizes

To ascertain the association, the cumulative risk ratio (RR) along with its corresponding 95% confidence interval was calculated utilizing the meta set command. This calculation incorporated logarithmic transformation, as well as the logarithmic standard deviation of the risk ratio. Heterogeneity among the included studies was assessed through the I2 statistic and the Q Cochrane test. Following Cochrane's criteria, I2 values of 0 to 25% were indicative of no heterogeneity, 25 to 50% suggested low heterogeneity, 50 to 75% indicated high but acceptable heterogeneity, and 75 to 100% pointed to high and unacceptable heterogeneity.

To evaluate potential publication bias, a funnel plot and the Egger test were employed. The statistical analyses were conducted using STATA 17.0, and a significance threshold of *P* < 0.05 was considered for all assessments.

## Results

### Study selection

**Fig. 1:**
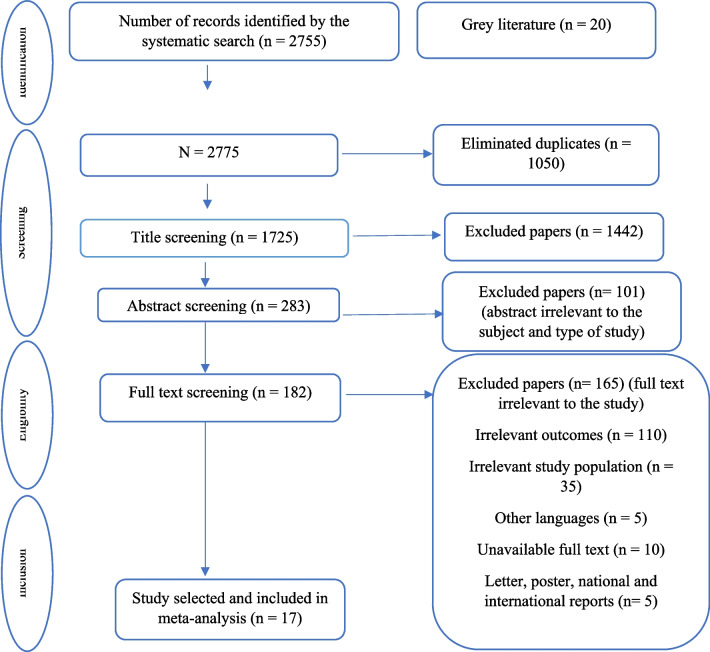
The search outputs and study selection

At the end of the search, 2775 articles were retrieved from the reviewed databases, of which 1050 were duplicates, and were excluded from the study. After screening by title, 1442 articles were removed out of 1725 ones, and 283 studies were evaluated based on their abstracts. Then, 182 articles were entered into the screening stage based on their full texts, of which 110 ones were excluded from the study due to different outcomes, 35 due to different communities, 5 due to non-English language, 10 due to unavailability, and 5 due to different methods. Finally, 17 clinical trial studies were included (Fig. 1).

### Study characteristics and results of individual studies

Among all selected articles, 15 studies were randomized clinical trials, and 2 ones were non-randomized clinical trials. Also, 6 studies were published in 2020, and the rest in 2021. In the final 11 selected studies, the comparison group received a placebo while in the rest of the studies, they received another drug, such as dexamethasone, favipiravir, or sarilumab. The mean age and BMI in all studies was 61.4 years, and 27.7 kg/m^2^, respectively (Table 1). In seven studies, the tocilizumab consumption increased the risk of mortality, and the control group of two studies received only the usual treatment of COVID-19 without additional drugs, or placebo [22, 23]. The control group of three studies received placebo in addition to the usual treatment of COVID-19 [24–26]. Another study compared the effects of dexamethasone, and tocilizumab [27] while dexamethasone was prescribed at a dose of 4 mg/kg/day, and was more effective than tocilizumab in preventing death due to the COVID-19 disease. Finally, the last study examined the effect of the usual dose of tocilizumab on the first group receiving doses of 200, and 120 mg, and the second group receiving doses of 80, and 40 mg. It is likely that the higher mortality in the first group than the second one is due to sicker patients, and the prescription of higher doses of the drug for them [28]. Among the series of articles related to reduction in mortality, the study by Capra et al. showed the most significant rate [29] (Table 1). Among the ten articles in which the use of tocilizumab reduced mortality, in one study, the control group received favipiravir [30], in another study, the two separate control groups received sarilumab, and placebo [31], in one study, they received methylprednisolone [32], and in five studies, the control groups received the usual treatment of COVID-19 [33–36]. In two studies, grouping was performed based on the response [37], and disease severity [38] (Table 1).

**Table 1:** The characteristics of included studies

Authors	Countries Years	Type of study	Total sample size	Sample size (intervention)	Sample size (comparison	No. of death in intervention group	No. of death in placebo group	Comparison	Age	BMI
V. C. Veiga, et al^22^	Brazil 2021	RCT	129	65	64	30	9	Placebo	57.5	
I. O. Rosas, et al^23^	UK 2021	RCT	438	294	144	57	27	Placebo	60.8	
D. Wang, et al^24^	USA 2020	RCT	65	34	31	2	4	Placebo	63	
R. Capra, et al^25^	Italy 2020	RCT	85	62	23	2	11	Placebo	65	
A. Rashad, et al^26^	Egypt 2021	RCT	149	74	75	60	45	Dexamethasone	62.3	
O. Hermine, et al^27^	France 2021	RCT	130	63	67	7	8	Placebo	63.7	27.7
A. S. Soin, et al^28^	India 2021	RCT	180	91	89	11	15	Placebo	55	26.9
Perrone, F., et al^29^	Italy 2020	RCT	1221	301	920	8	11	Placebo		
J. H. Stone, et al^30^	UK 2020	RCT	242	161	81	9	3	Placebo	59.8	30.1
D. M. Hamed, et al^31^	United Arab Emirates 2021	RCT	76	49	27	3	5	Difference in dosage	48	
Hong Zhao, et al^32^	China 2021	RCT	26	14	7	0	2	Favipiravir	75	24.8
G. W. Strohbehn, et al^33^	UK 2021	CT	32	12	20	2	3	Difference in dosage	69	
E. H. Baker, et al^34^	UK 2021	RCT	17	11	6	2	0	Placebo		
A. C. Gordon, et al^35^	UK 2021	RCT	755	353	402	99	142	Placebo	61.4	
A. C. Gordon, et al^35^	UK 2021	RCT	450	48	402	10	142	Sarilumab	61.4	
F. Dastan, et al^36^	Iran 2020	CT	42	20	22	1	6	Placebo	56	
G. Pomponio, et al^37^	Italy 2020	RCT	46	25	21	7	0	Placebo	66.5	
C. Salama, et al^38^	United States, Mexico, Kenya, South Africa, Peru, or Brazil 2021	RCT	388	259	129	26	11	Placebo	55.9	32.4

### Results of synthesis

**Fig. 2:**
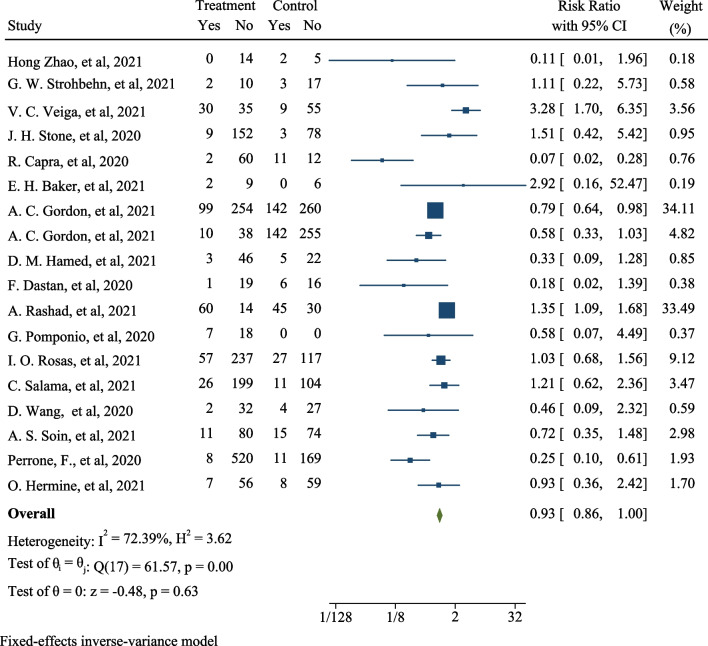
The effect of tocilizumab (Actemra) on the occurrence of death in patients with COVID-19

**Fig. 3:**
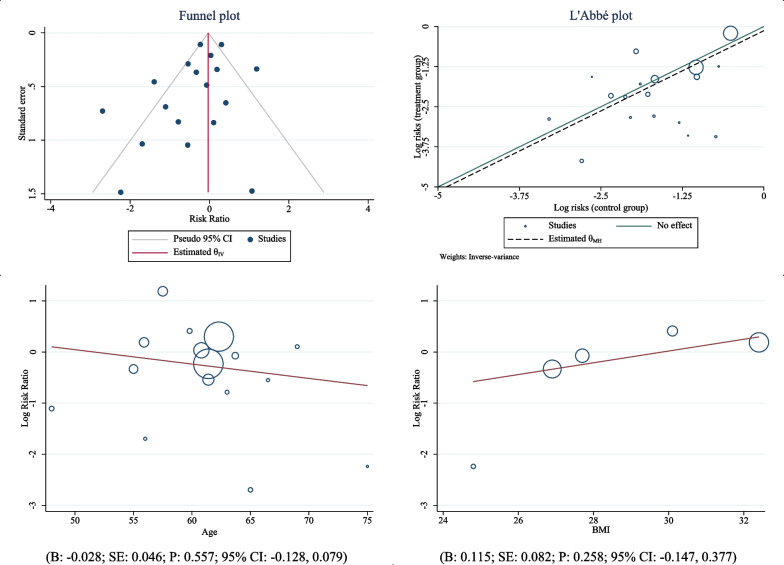
The funnel plot (for assessing heterogeneity) and L’Abbe plot (for assessing heterogeneity)

After combining the results of these studies, the cumulative risk of death in COVID-19 patients taking tocilizumab was 0.93 compared to patients who had not taken the drug (RR: 0.93; 95% CI: 0.86, 1.00; *I*^2^: 72.39%) (Fig. 2). The results of publication bias using the Egger test and funnel plot showed publication bias did not occur in this analysis (*B*: − 1.08; standard _Error_: 0.608; *P*-value: 0.075). The funnel plot is shown in Fig. 3. The percentage of heterogeneity in this analysis was 72.39% which was less than 75%, and indicates the presence of heterogeneity between studies, but its rate is acceptable (Fig. 3). In addition to these findings, meta-regression results are shown in Fig. 3. This analysis was performed to evaluate the effect of patients’ body mass index, and age on the association between the use of tocilizumab, and the rate of death due to COVID-19. The results showed with increasing age, the effect of this drug on reducing death due to COVID-19 in patients decreased (*B*: − 0.028; Standard _Error_: 0.046; *P*-value: 0.557; 95% CI: − 0.128, 0.079), and with increasing the body mass index, the effect of tocilizumab on reducing death caused by COVID-19 in patients increased (*B*: 0.115; Standard _Error_: 0.082; *P*-value: 0.258; 95% CI: − 0.147, 0.377), but the association between both variables (the age, and body mass index) was not statistically significant in meta-regression analysis (Fig. 3).

**Fig. 4:**
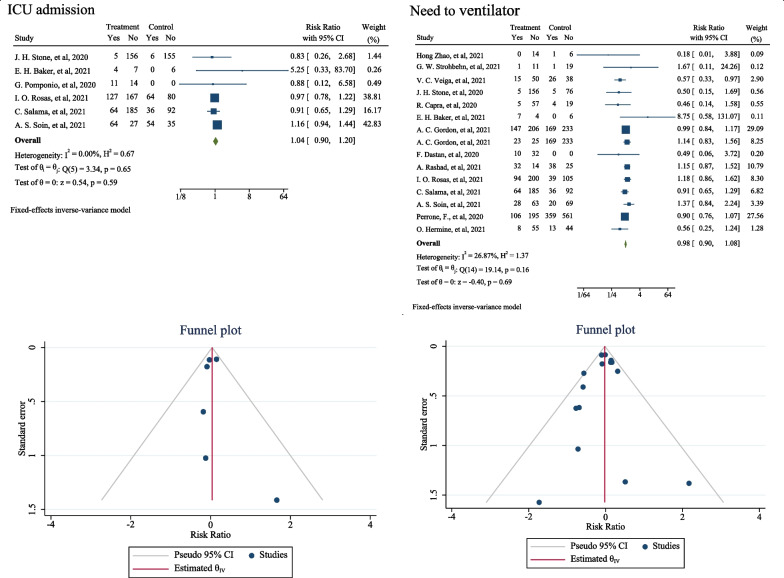
The effect of tocilizumab (Actemra) on the occurrence of ICU admission and need to ventilator in patients with COVID-19 (forest and funnel plot)

In addition to death, the outcomes of the need for a ventilator, and hospitalization in ICU were also examined, the results of which are shown in Fig. 4. The results showed tocilizumab consumption increased hospitalization in ICU by 4% which was not statistically significant (RR: 1.04; 95% CI: 0.90, 1.20; I _square_: 0.00%) (Fig. 4). The results of the present meta-analysis also showed the use of this drug reduced the need for a ventilator by 2%, but this association was also not statistically significant (RR: 0.98; 95% CI: 0.90, 1.08; I _square_: 26.87%) (Fig. 4). The results of publication bias analysis using the Egger test, and funnel plot showed publication bias did not occur in any of these associations (*B*: -0.36; Standard _Error_: 0.400; *P*-value: 0.376) (*B*: 0.13; Standard _Error_: 0.657; *P*-value: 0.840). The funnel plot is shown in Fig. 4. In addition, meta-regression was performed to evaluate the effect of COVID-19 patients’ body mass index, and age on the association between the use of tocilizumab, hospitalization in ICU, and the need for ventilator. The results showed with increasing age, and the body mass index, increased the effect of tocilizumab on the need for a ventilator in patients with COVID-19 ((*B*: 0.001; standard _Error_: 0.027; *P*-value: 0.950; 95% CI: − 0.059, 0.067), and (*B*: 0.006; standard _Error_: 0.116; *P*-value: 0.960; 95% CI: − 0.36, 0.37)). Also, these variables lead to decreased the effect of tocilizumab on hospitalization of patients with COVID-19 in ICU ((*B*: − 0.028; standard _Error_: 0.025; *P*-value: 0.425; 95% CI: -0.105, 0.057), and (*B*: − 0.040; standard _Error_: 0.037; *P*-value: 0.447; 95% CI: -0.52, 0.43)).

In this meta-analysis, the association between underlying diseases, severe forms of the COVID-19 disease, and the use of tocilizumab was investigated, and the results have been shown in Table 2. The results showed hypertension, cardiovascular diseases, asthma, malignancy, chronic pulmonary disorders, chronic liver disorders, and obesity could make patients with COVID-19 more prone to its severe forms, and taking tocilizumab. Among the mentioned diseases, the chronic liver disorder could have a greater effect, but this association was not statistically significant (RR: 1.43; 95% CI: 0.42, 4.86; *I*^2^: 0.00%) (Table 2). Only hypertension was significantly associated with the severe form of the COVID-19 disease in patients, and tocilizumab consumption (RR: 1.03; 95% CI: 1.00, 1.12; *I*^2^:: 28.08%) (Table 2). In addition, myocardial infraction, diabetes mellitus, COPD, and chronic kidney disorders reduced the risk of developing severe COVID-19 forms, and the use of tocilizumab according to the combination of preliminary study results (Table 2).

**Table 2:** The effect of presence non-communicable diseases on the prescript of tocilizumab in patients with COVID-19

Variables	Subgroups (no. of studies/sample size)	Pooled effect size (% 95 CI)			Heterogeneity assessment		
		RR	Lower CI	Upper CI	*I* square (%)	*Q* Test	*P* value
Non-communicable diseases	Hypertension	1.03	1.00	1.12	28.08	11.12	0.19
	Diabetes mellitus	0.83	0.72	0.95	0.00	7.00	0.54
	Obesity	1.07	0.76	1.50	0.00	0.32	0.57
	Cardiovascular disease	1.17	0.98	1.40	20.43	8.80	0.27
	Myocardial infraction	0.69	0.32	1.45	81.99	5.55	0.02
	COPD	0.88	0.43	1.80	0.00	1.61	0.66
	Asthma	1.27	0.65	2.50	0.08	3.00	0.39
	Chronic kidney disorder	0.97	0.63	1.50	25.59	5.38	0.25
	Malignancy	1.14	0.65	2.00	0.00	0.82	0.84
	Chronic pulmonary disorder	1.20	0.71	2.03	0.00	0.02	0.99
	Chronic liver disorder	1.43	0.42	4.86	0.00	0.00	0.96

### Risk of bias results

**Fig. 5:**
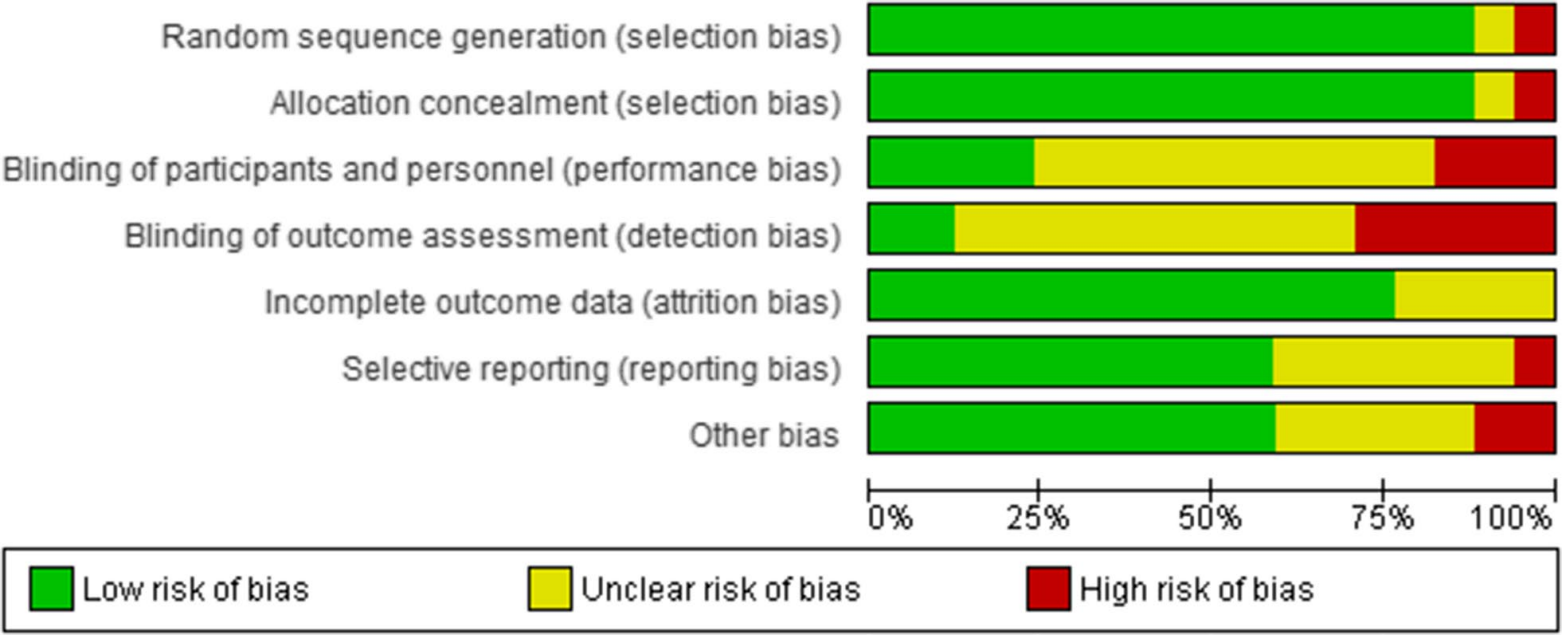
Risk of bias graph: review authors' judgements about each risk of bias item presented as percentages across all included studies

The qualitative assessment of articles based on the Cochrane checklist revealed that the initially selected studies exhibited a low risk of bias. Notably, the least bias was observed in the areas of allocation concealment and random sequence generation, indicating minimal risk of selection bias (see Fig. 5). Conversely, the most prevalent bias was associated with the blinding of outcome assessment, indicative of detection bias, with several initial studies manifesting challenges in this domain (see Fig. 5).

**Fig. 6:**
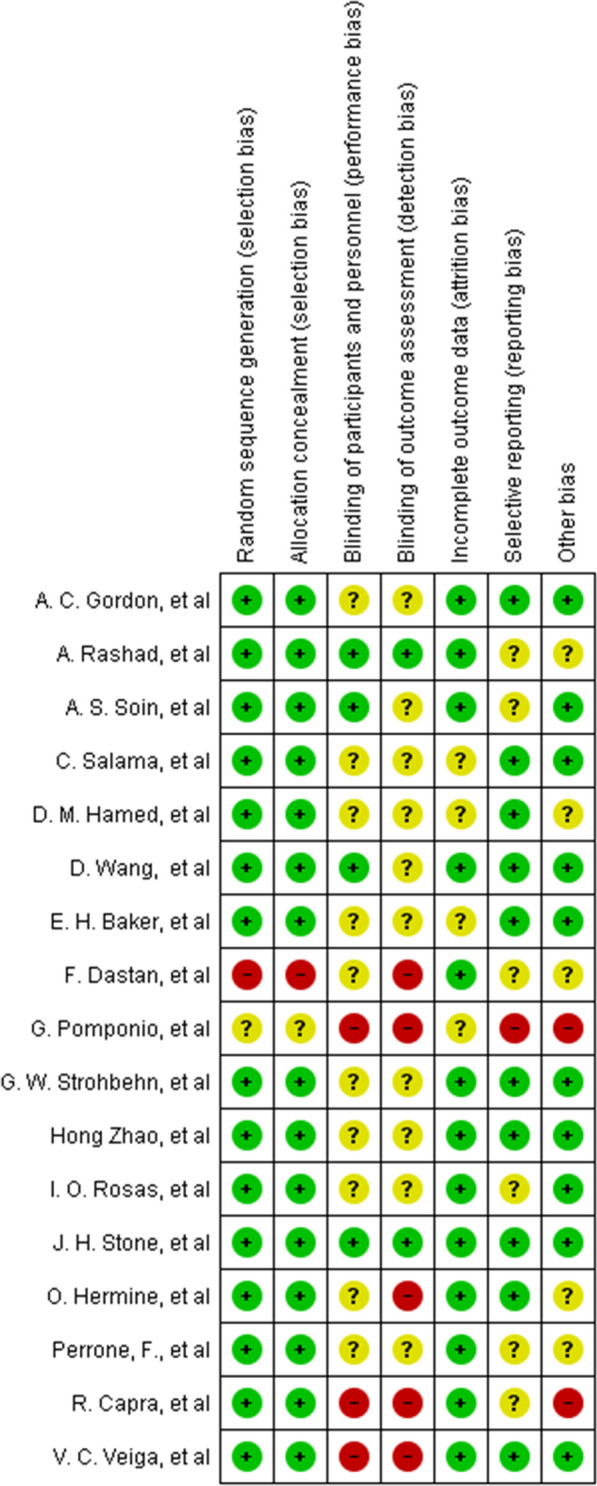
Risk of bias summary: review authors' judgements about each risk of bias item for each included study

Within the selected studies, the study conducted by Stone et al. exhibited the least bias, whereas the study by Pomponio et al. demonstrated the highest level of bias (see Fig. 6). These findings underscore the importance of considering specific domains of bias in the individual studies, allowing for a nuanced understanding of the methodological rigor and limitations across the body of evidence.

## Discussion

The primary objective of this meta-analysis was to assess the impact of tocilizumab on outcomes associated with COVID-19. The findings indicate a 7% reduction in the mortality rate among patients with COVID-19 treated with tocilizumab. Additionally, the administration of this drug was associated with a 4% increase in ICU hospitalization and a 2% reduction in the need for mechanical ventilation; however, these associations did not reach statistical significance in this meta-analysis. Tocilizumab, classified among IL-6 inhibitors, is recognized for its potential in managing acute respiratory distress syndrome (ARDS) induced by cytokine storms in affected patients. Functioning as a recombinant human monoclonal antibody, tocilizumab binds to both the soluble IL-6 receptor and the membrane-bound IL-6 receptor. These mechanistic characteristics underscore its role in modulating the inflammatory response, particularly in the context of severe respiratory complications associated with COVID-19 [39]. Originally employed in the treatment of rheumatoid arthritis and systemic juvenile idiopathic arthritis, this drug was chosen for its anti-inflammatory effects. Its mechanism of action in rheumatoid arthritis, akin to the pathophysiology of COVID-19, centers on its ability to mitigate the impact of elevated interleukin-6 (IL-6) levels. In patients with rheumatoid arthritis, characterized by a substantial volume of IL-6 in both synovial fluid and serum, tocilizumab selectively binds to this cytokine, consequently alleviating the symptomatic manifestations of the disease. This shared mechanism of action underlines the rationale for exploring tocilizumab's efficacy in the context of COVID-19, given the inflammatory nature of the latter condition [40]. According to a multicenter study conducted by Toniati et al., the prescription of tocilizumab reduced the occurrence of ARDS failure in these patients [41]. Also, based on the natural history of the COVID-19 disease, one of the processes involved in the occurrence of its critical, and severe cases is the deregulation of the patient's immune system [42]. The COVID-19 disease engages both innate and acquired immunity. Effective control of the immune response against the disease relies on the secretion of various pro-inflammatory cytokines. Additionally, activation of T cells plays a crucial role, as their function involves regulating viral replication, inhibiting viral spread, limiting inflammation, and clearing infected cells. The coordination between innate and acquired immunity, coupled with the orchestrated release of pro-inflammatory mediators and the involvement of T cells, is essential for a robust and comprehensive defense mechanism against the viral infection [43, 44]. Immune system activity seems to be effective in the progress of lung injuries in patients [45]. The SARS-CoV-2 virus triggers the activation of T helper 1 cells, leading to the secretion of pro-inflammatory agents such as granulocyte–macrophage colony-stimulating factor (GM-CSF) and interleukin-6 (IL-6). Notably, GM-CSF is instrumental in activating inflammatory monocytes, specifically CD14 + and CD16 + subsets, which in turn produce substantial quantities of IL-6, tumor necrosis factor-alpha (TNF-α), and various other cytokines. This orchestrated immune response underscores the complex interplay between T helper cells, GM-CSF, and inflammatory monocytes in generating a robust pro-inflammatory milieu during SARS-CoV-2 infection [46]. Published articles suggest that high levels of IL-6 significantly increase the risk of hospitalization in ICU, and developing ARDS, and cause death. Patients with a critical condition of the COVID-19 disease have higher serum IL-6 levels [47]. In fact, studies show high levels of pro-inflammatory cytokines, TNF-α, and IL-6 are involved in the occurrence of cytokine storms [46]. Cytokine storm is a general term characterized by clinical symptoms, systemic inflammation, and ultimately dysfunction of several organs, and if left untreated, it can lead to failure of several organs [48]. CRS manifests as uncontrolled systemic inflammation, characterized by the widespread activation of macrophages, dendritic cells (DCs), natural killer (NK) cells, B cells, and T cells. CRS is marked by the excessive production of elevated levels of pro-inflammatory cytokines, including TNFα, IL1β, IL6, IL12, IL18, IL33, IFN-I, and IFNγ, alongside chemokines such as CCL2, CCL3, CCL5, CXCL8, CXCL9, and CXCL10. This syndrome can arise in various conditions, encompassing adoptive T cell therapy, graft-versus-host disease (GvHD), and systemic infections. The systemic and multifaceted nature of CRS underscores its potential impact on immune homeostasis and its association with diverse pathological states [49].

Cytokine storms can manifest with a spectrum of symptoms, ranging from mild to severe and potentially life-threatening. Mild symptoms of CRS encompass fever, fatigue, rash, arthralgia, and myalgia. In more severe cases, individuals may experience hypotension, high fever, and an uncontrolled systemic response that can lead to shock and multiple organ failure. Respiratory symptoms are particularly common in patients with CRS, ranging from mild manifestations such as cough and tachypnea to severe conditions like ARDS. The diverse clinical presentation underscores the variability and potential severity of CRS across affected individuals [50]. ARDS is a non-cardiogenic pulmonary edema, which presents as rapidly progressing shortness of breath, tachypnea, and hypoxemia. ARDS is more likely to occur when a pulmonary, or extra-pulmonary accident triggers the release of inflammatory mediators, resulting in the accumulation of inflammatory cells in the alveoli, and pulmonary microcirculation [51, 52]. Therefore, due to the nature of the disease, and the effect of the drug, this medicine inhibits the accumulation of pro-inflammatory cytokines, and ultimately reduces the expected outcomes in COVID-19, such as death.

In a similar meta-analysis focusing on data collection from ten clinical trials, the effect of tocilizumab on reducing mortality as well as reducing the need for mechanical ventilation in patients was observed. The results of this article also confirmed tocilizumab might be involved in reducing secondary complications such as disease exacerbation, hospitalization in ICU, and composite [53]. These results were in line with the ones of the present meta-analysis. A systematic review, and meta-analysis was conducted by Aziz et al. on the effect of tocilizumab on COVID-19 in 2021. In this study, a total of 23 articles which had examined 6279 patients were evaluated. According to the results of the study, the intervention group who received tocilizumab showed a reduction in mortality, and the need for a mechanical ventilator compared to the control group who received standard care [15]. In another meta-analysis conducted by Mahroum et al. in 2021, 39 case–control studies with a population of 15,531 patients were included. Finally, the adjusted data confirmed the effect of tocilizumab on reducing mortality, and also preventing patients from being admitted to ICU[54]. The results of a meta-analysis including 13 articles, and 766 patients, performed by Kotak et al. to evaluate the safety, and efficacy of tocilizumab in patients with COVID-19 in 2020 showed a significant effect of the drug on reducing mortality in patients. Although no significant effect was observed on the rate of hospitalization in ICU, and infections after treatment, the percentage of oxygen saturation in these patients showed a significant improvement [55]. This meta-analysis distinguishes itself from previously published ones examining the impact of tocilizumab on COVID-19-related mortality by exclusively focusing on clinical trial studies, in contrast to many past analyses that incorporated retrospective or prospective studies. Notably, the present analysis maintains a lower level of heterogeneity, meeting acceptable criteria according to Cochrane standards, unlike previous meta-analyses that exhibited higher heterogeneity. Furthermore, the current meta-analysis surpasses its predecessors by incorporating a larger number of articles and a higher overall sample size, enhancing the precision of the effect size estimation for tocilizumab's role in reducing mortality. The analysis also delves into the risk of chronic, non-communicable diseases in COVID-19 patients receiving tocilizumab, a facet largely unexplored in prior meta-analyses. This study extends its scope by investigating the drug's impact on hospitalization in intensive care units and the need for ventilators, outcomes that have been previously explored, yet not consistently reported. Given that tocilizumab is primarily prescribed for COVID-19 patients prone to ICU admission and ventilator use, caution is warranted in interpreting its effects on these outcomes. Nevertheless, notable limitations include insufficient information regarding the drug's effect on mortality considering variables such as underlying diseases, age, body mass index, hospitalization duration, and concurrent medication use. To ascertain the drug's genuine effects on COVID-19 patients, future cohort studies with ample sample sizes are strongly recommended, particularly focusing on mortality in relation to the mentioned variables.

## Conclusion

The administration of tocilizumab during the COVID-19 pandemic, when prescribed to patients with the virus, demonstrated a notable reduction in the incidence of patient mortality. Nevertheless, for a more precise understanding, it is imperative to conduct cohort studies with a substantial sample size, focusing on COVID-19 patients undergoing this treatment. These studies should specifically evaluate the long-term effects of tocilizumab, considering crucial influencing variables such as the presence of underlying diseases and various demographic factors. A comprehensive examination of these factors will contribute to a more nuanced and comprehensive assessment of tocilizumab's efficacy and safety in the context of COVID-19 treatment.

## Data Availability

The datasets used and analyzed during the current study are available from the corresponding author on reasonable request.

## Abbreviations

SARS-CoV-2: Severe acute respiratory syndrome coronavirus-2
WHO: World Health Organization
CRS: Cytokine release syndrome
ICU: Intensive care unit
PRISMA: The Preferred Reporting Items for Systematic Reviews and Meta-Analyses statement
CONSORT: The Consolidated Standards of Reporting Trials Statement
RR: Risk ratio
CI: Confidence interval
ARDS: Acute respiratory distress syndrome
GM-CSF: Granulocyte–macrophage colony-stimulating factor
IL-6: Interleukin-6

## References

[1] Wu A (2020). Genome composition and divergence of the novel coronavirus (2019-nCoV) originating in China. Cell Host Microbe.

[2] Organization, W.H., *World Health Organization Coronavirus Disease (COVID-19): Weekly Epidemiological Update*. 2021.

[3] Phelan AL, Katz R, Gostin LO (2020). The novel coronavirus originating in Wuhan, China: challenges for global health governance. JAMA.

[4] Zhang C (2020). Cytokine release syndrome in severe COVID-19: interleukin-6 receptor antagonist tocilizumab may be the key to reduce mortality. Int J Antimicrob Agents.

[5] Coperchini F (2020). The cytokine storm in COVID-19: An overview of the involvement of the chemokine/chemokine-receptor system. Cytokine Growth Factor Rev.

[6] Tanaka T, Narazaki M, Kishimoto T (2014). IL-6 in inflammation, immunity, and disease. Cold Spring Harb Perspect Biol.

[7] Laguna-Goya, R., et al., *IL-6–based mortality risk model for hospitalized patients with COVID-19.* Journal of Allergy and Clinical Immunology, 2020. **146**(4): p. 799–807. e9. 10.1016/j.jaci.2020.07.009 PMC7375283 32710975

[8] Singh JA, Beg S, Lopez-Olivo MA (2011). Tocilizumab for rheumatoid arthritis: a Cochrane systematic review. J Rheumatol.

[9] Rubbert-Roth A (2018). A review of recent advances using tocilizumab in the treatment of rheumatic diseases. Rheumatology and therapy.

[10] Lee DW (2014). Current concepts in the diagnosis and management of cytokine release syndrome. Blood, The Journal of the American Society of Hematology.

[11] Tleyjeh IM (2021). Efficacy and safety of tocilizumab in COVID-19 patients: a living systematic review and meta-analysis, first update. Clin Microbiol Infect.

[12] Guaraldi G (2020). Tocilizumab in patients with severe COVID-19: a retrospective cohort study. The Lancet Rheumatology.

[13] Charan J (2021). Tocilizumab in COVID-19: a study of adverse drug events reported in the WHO database. Expert Opin Drug Saf.

[14] Tleyjeh IM (2021). Efficacy and safety of tocilizumab in COVID-19 patients: a living systematic review and meta-analysis. Clin Microbiol Infect.

[15] Aziz M (2021). Efficacy of tocilizumab in COVID-19: a systematic review and meta-analysis. J Med Virol.

[16] Piscoya A (2022). Efficacy and harms of tocilizumab for the treatment of COVID-19 patients: A systematic review and meta-analysis. PLoS ONE.

[17] Della-Torre E (2020). Interleukin-6 blockade with sarilumab in severe COVID-19 pneumonia with systemic hyperinflammation: an open-label cohort study. Ann Rheum Dis.

[18] Page MJ (2021). The PRISMA 2020 statement: an updated guideline for reporting systematic reviews. Syst Rev.

[19] Moher, D., et al., *The CONSORT statement: revised recommendations for improving the quality of reports of parallel-group randomised trials*. 2001, Elsevier. 11323066

[20] Schulz KF, Altman DG, Moher D (2010). CONSORT 2010 statement: updated guidelines for reporting parallel group randomised trials. BMC Med.

[21] Higgins JP (2011). The Cochrane Collaboration’s tool for assessing risk of bias in randomised trials. BMJ.

[22] Veiga, V.C., et al., *Effect of tocilizumab on clinical outcomes at 15 days in patients with severe or critical coronavirus disease 2019: randomised controlled trial.* bmj, 2021. **372**. 10.1136/bmj.n84 PMC7815251 33472855

[23] Rosas, I.O., et al., *Tocilizumab in hospitalized patients with COVID-19 pneumonia.* MedRxiv, 2020.

[24] Wang, D., et al., *Tocilizumab in patients with moderate or severe COVID-19: a randomized, controlled, open-label, multicenter trial. Front Med 2021.[Epub ahead of print]*. PUBMED. 10.1007/s11684-020-0824-3 PMC7940448 33687643

[25] Capra R (2020). Impact of low dose tocilizumab on mortality rate in patients with COVID-19 related pneumonia. Eur J Intern Med.

[26] Rashad A (2021). Short term survival of critically ill COVID-19 Egyptian patients on assisted ventilation treated by either Dexamethasone or Tocilizumab. Sci Rep.

[27] Hermine O (2021). Effect of tocilizumab vs usual care in adults hospitalized with COVID-19 and moderate or severe pneumonia: a randomized clinical trial. JAMA Intern Med.

[28] Soin AS (2021). Tocilizumab plus standard care versus standard care in patients in India with moderate to severe COVID-19-associated cytokine release syndrome (COVINTOC): an open-label, multicentre, randomised, controlled, phase 3 trial. Lancet Respir Med.

[29] Perrone, F., et al., *Tocilizumab for patients with COVID-19 pneumonia. The single-arm TOCIVID-19 prospective trial.* Journal of translational medicine, 2020. **18**(1): p. 1–11. 10.1186/s12967-020-02573-9 PMC7576974 33087150

[30] Rocco, P.R., et al., *Early use of nitazoxanide in mild Covid-19 disease: randomised, placebo-controlled trial.* European Respiratory Journal, 2021. **58**(1). 10.1183/13993003.03725-2020 PMC7758778 33361100

[31] Hamed DM (2021). Intravenous methylprednisolone with or without tocilizumab in patients with severe COVID-19 pneumonia requiring oxygen support: A prospective comparison. J Infect Public Health.

[32] Zhao H (2021). Tocilizumab combined with favipiravir in the treatment of COVID-19: A multicenter trial in a small sample size. Biomed Pharmacother.

[33] Strohbehn GW (2021). COVIDOSE: a phase II clinical trial of low-dose tocilizumab in the treatment of noncritical COVID-19 pneumonia. Clin Pharmacol Ther.

[34] Baker, E.H., et al., *Insights from compassionate use of tocilizumab for COVID‐19 to inform appropriate design of randomised controlled trials.* British Journal of Clinical Pharmacology, 2021. 10.1111/bcp.14466 PMC7405226 32656822

[35] McAuley, D. and R.-C.W. Committee, *Interleukin-6 Receptor Antagonists in Critically Ill Patients with Covid-19.* New England Journal of Medicine, 2021. 10.1056/NEJMc2108482 34407334

[36] Dastan F (2020). Promising effects of tocilizumab in COVID-19: a non-controlled, prospective clinical trial. Int Immunopharmacol.

[37] Pomponio G (2021). Tocilizumab in COVID-19 interstitial pneumonia. J Intern Med.

[38] Salama, C. and S.V. Mohan, *Tocilizumab in patients hospitalized with Covid-19 pneumonia. Reply.* The New England journal of medicine, 2021. 10.1056/NEJMc2100217 33657287

[39] Khiali S, Khani E, Entezari-Maleki T (2020). A comprehensive review of tocilizumab in COVID-19 acute respiratory distress syndrome. J Clin Pharmacol.

[40] Oldfield V, Dhillon S, Plosker GL (2009). Tocilizumab. Drugs.

[41] Toniati P (2020). Tocilizumab for the treatment of severe COVID-19 pneumonia with hyperinflammatory syndrome and acute respiratory failure: a single center study of 100 patients in Brescia, Italy. Autoimmun Rev.

[42] Anka AU (2021). Coronavirus disease 2019 (COVID-19): An overview of the immunopathology, serological diagnosis and management. Scand J Immunol.

[43] Li G (2020). Coronavirus infections and immune responses. J Med Virol.

[44] Ivashkiv LB, Donlin LT (2014). Regulation of type I interferon responses. Nat Rev Immunol.

[45] Khosroshahi LM (2021). Immunology, immunopathogenesis and immunotherapeutics of COVID-19; an overview. Int Immunopharmacol.

[46] Hu B, Huang S, Yin L (2021). The cytokine storm and COVID-19. J Med Virol.

[47] Coomes EA, Haghbayan H (2020). Interleukin-6 in COVID-19: a systematic review and meta-analysis. Rev Med Virol.

[48] Gaestel, M., A.R. Nebreda, and M.B. Yaffe, *Cytokine Storm.* 2021. 10.1056/NEJMc2036236 33882214

[49] Tan M (2020). Immunopathological characteristics of coronavirus disease 2019 cases in Guangzhou. China Immunology.

[50] Shimabukuro-Vornhagen A (2018). Cytokine release syndrome. J Immunother Cancer.

[51] Thompson BT, Chambers RC, Liu KD (2017). Acute respiratory distress syndrome. N Engl J Med.

[52] Matthay MA (2019). Acute respiratory distress syndrome. Nat Rev Dis Primers.

[53] Snow TAC (2021). Tocilizumab in COVID-19: a meta-analysis, trial sequential analysis, and meta-regression of randomized-controlled trials. Intensive Care Med.

[54] Mahroum N (2021). Systematic review and meta-analysis of tocilizumab therapy versus standard of care in over 15,000 COVID-19 pneumonia patients during the first eight months of the pandemic. Int J Environ Res Public Health.

[55] Kotak, S., et al., *Use of tocilizumab in COVID-19: a systematic review and meta-analysis of current evidence.* Cureus, 2020. **12**(10). 10.7759/cureus.10869 PMC7652362 33178522

